# Removal of Butyl Mercaptan from Gas Streams by Reactive Adsorption

**DOI:** 10.3390/molecules30091962

**Published:** 2025-04-28

**Authors:** Mia Sanda, Ion Onuțu, Cristina Maria Dușescu-Vasile, Gabriel Vasilievici, Dorin Bomboș, Marian Băjan, Gheorghe Brănoiu

**Affiliations:** 1 Department of Petroleum Refining Engineering and Environmental Protection, Petroleum-Gas University of Ploiesti, 39 Bucharest Blvd., 100680 Ploiesti, Romania; mia.sanda@yahoo.com (M.S.); ionutu@upg-ploiesti.ro (I.O.); marian.bajan@upg-ploiesti.ro (M.B.); 2 National Institute for Research Development for Chemistry and Petrochemistry-ICECHIM-București, 202 Spl. Independenței, 060021 Bucharest, Romania; gvasilievici@icechim.ro; 3 Department of Chemistry, Petroleum-Gas University of Ploiesti, 39 Bucharest Blvd., 100680 Ploiesti, Romania; 4 Department of Well Drilling, Extraction and Transport of Hydrocarbons, Petroleum-Gas University of Ploiesti, 39 Bucharest Blvd., 100680 Ploiesti, Romania; gheorghe.branoiu@upg-ploiesti.ro

**Keywords:** butyl mercaptan, reactive adsorption, activated carbon, oxides of Cu, Fe and Zn

## Abstract

1-butanethiol, a volatile mercaptan that is harmful and has a persistent odor, was adsorbed from a gaseous stream onto granulated activated carbon (AC) that was doped with Cu, Fe, and Zn oxides. The adsorbents were prepared by precipitating salts of the respective metals using an ammonia solution, along with the inclusion of an anti-caking agent known as Pluronic-123. Characterization of the three prepared adsorbents was conducted using electron microscopy (SEM), textural analysis, thermogravimetric analysis, FTIR, and XRD. The study’s results indicate that the adsorbents exhibit different textural characteristics and variations in the size and shape of the metal oxide clusters deposited on the activated carbon. These differences also led to variations in the adsorption capacity for 1-butanethiol among the three adsorbents.

## 1. Introduction

Hydrogen sulfide and its organic derivatives, such as lower mercaptans, are volatile and toxic compounds with an unpleasant odor that can pollute the air. These compounds may be produced or utilized in various industrial processes [1]. For instance, hydrogen sulfide can be generated during the production of carbon disulfide, in the hydro-refining of sulfur-containing petroleum products, and in the manufacture of viscose, among other processes [2,3]. The health effects associated with these pollutants include headaches, nausea, low blood pressure, loss of appetite, weight loss, ataxia, inflammation of the ocular membrane, and chronic cough [4,5,6]. Mercaptans, known for their low olfactory thresholds and foul odors, pose challenges to industrial processes and human health. Among the various methods for treating mercaptans, adsorption has been extensively studied and implemented due to its high efficiency and the regenerability of adsorbents [7,8,9,10]. Porous carbon nanofibers have been used for the adsorption and removal of volatile organic compounds. The introduction of graphene oxide onto composite carbon nanofibers with well-developed mesoporous structures simultaneously improve the adsorption capacities and adsorption tendency of nanofibers for polar VOCs [11].

Various processes have been developed for the depollution of gaseous streams containing mercaptans. For instance, mercaptans can be effectively adsorbed onto hexagonal boron nitride (h-BN) nanosheets that are doped with metals such as cobalt (Co), copper (Cu), iron (Fe), and nickel (Ni). Thus, research has shown that doping these nanosheets with metals enhances the adsorption process by reducing the adsorption energies. It has been noted that the adsorption energy increases with the length of the alkyl chain in the mercaptan molecules [12]. Additionally, porous carbon derived from rice husks has proven to be an efficient adsorbent for treating odorous gases that contain sulfur compounds in the air, successfully removing ethyl mercaptan. The high adsorption capacity of this material can be attributed to its well-developed microporosity and the abundance of hydroxyl groups on its surface, which facilitate the chemisorption of ethyl mercaptan [13]. Furthermore, NaX zeolite exhibits a strong affinity for ethyl mercaptan adsorption, allowing it to preferentially capture this compound across a broad range of porosities [14]. Adsorbent materials designed for low concentrations of mercaptans in the air were also developed using activated carbon or metal–organic framework incorporated in a mesoporous silica, respectively [15,16,17]. The well-structured microporous characteristics of these adsorbents significantly enhance their efficiency in capturing methyl mercaptan (CH_3_SH).

In general, doping with metals or metal oxides is employed to enhance the selective adsorption capabilities of porous adsorbents. This enhancement occurs through interactions between the metal and certain methyl mercaptan molecules, which tend to ionize upon adsorption onto surfaces that feature functional groups containing nitrogen and oxygen. These functional groups are better at attracting counterions, thereby improving the adsorption performance of methyl mercaptan on activated carbon. For instance, a copper-modified SBA-15 adsorbent was utilized to remove odorous sulfur-containing pollutants, including methyl mercaptan. By increasing the dispersion of adsorption centers within the mesopore channels of SBA-15, the removal efficiency for CH_3_SH improved. For example, Peng et al. synthesized Cu-doped mesoporous silicas using Cu(NO_3_)_2_ at room temperature for 20 s to target the removal of low-concentration CH_3_SH. Compared with plain mesoporous silica, the mesoporous silica modified with 3% copper demonstrated a significantly longer saturation time of 118 min for CH_3_SH adsorption despite a decrease in surface area from 678.77 to 567.13 m²/g. The findings suggested that the surface groups on the CuO nanoparticles and the Si-O-Cu clusters likely transformed into a hydrated complex, making them more effective for capturing volatile organic compounds (VOCs). Initially, the porous adsorbents physically capture high-valence metal compounds, which are subsequently reduced by the active surface groups of the adsorbents. The strong affinity between reduced materials showing low valence and molecules of some VOCs can enhance the adsorption selectivity of porous adsorbents. In general, doping with metals/metal oxides changes the surface chemistry, along with the polarity of the porous adsorbent surface, so that the predominant adsorption mechanism changes from physical adsorption to chemical adsorption. Doping with metal species, such as metal salts (CaCl_2_, ZnCl_2_, FeCl_3_, Cu(NO_3_)_2_) and metal oxides (Fe_2_O_3_, MgO, CuO), is a common method for preparing adsorbents through metal deposition. This technique is widely applied for the adsorption of volatile organic compounds (VOCs), particularly in reactive adsorption processes [18]. The adsorbents are primarily prepared by impregnating them with solutions of metal salts, which significantly enhances the selective chemical adsorption of specific VOCs. However, the deposition of metal nanoparticles can lead to partial blockage of the surface in porous structures, resulting in a reduction in the available surface area and pore volume of the modified adsorbents. It is believed that doping with metals and metal oxides is particularly effective for the adsorption of VOCs at low concentrations, as there are fewer reaction centers available. The potential adsorption mechanism of specific VOC molecules on metal or metal oxide surfaces remains inadequately understood, highlighting the necessity for further investigation in this area. This study investigated the effectiveness of granulated activated carbon adsorbents doped with metal oxides—such as Cu, Fe, and Zn oxides—known for their high affinity for sulfur compounds. These adsorbents were prepared in the presence of a polymeric anti-clumping agent and tested for their ability to adsorb 1-butanethiol, a less-studied mercaptan with high persistence in the atmosphere.

## 2. Results and Discussion

### 2.1. SEM Analysis

**Figure 1:**
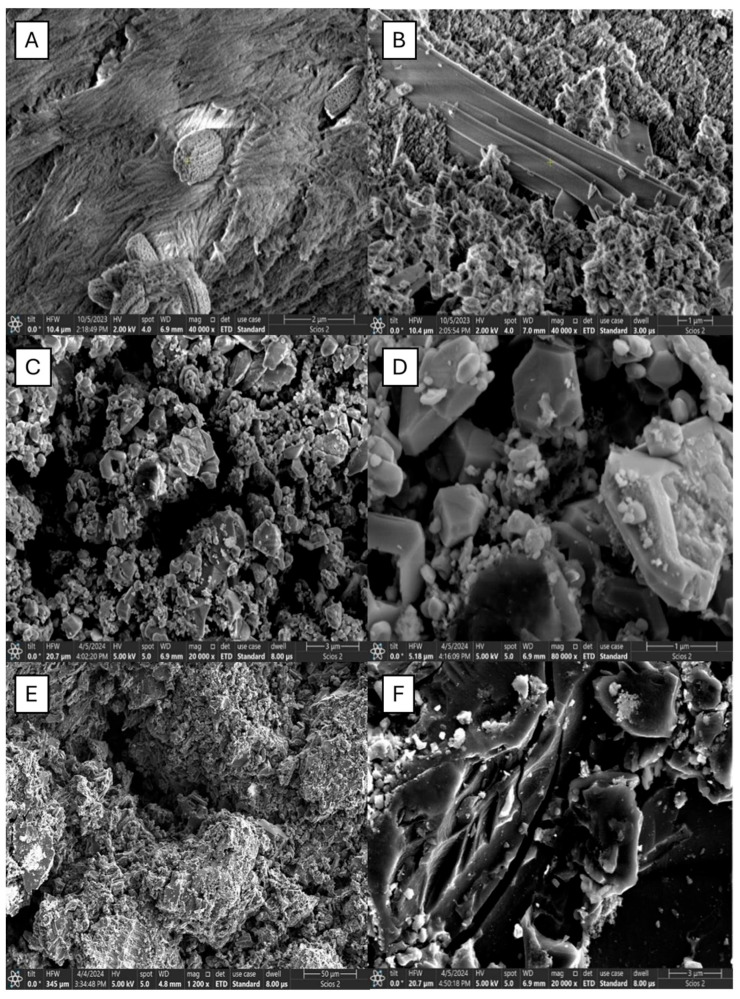
SEM images for the adsorbents, Cu/AC (**A**,**B**), Fe/AC (**C**,**D**), and Zn/AC (**E**,**F**).

The scanning electron microscopy analysis of adsorbents is presented in Figure 1. It is observed that the surface of the Cu/AC adsorbent is predominantly inhomogeneous. When you enlarge the image, you can see protuberances on a level surface and pores with different shapes, as well as particles of different diameters.

An obvious inhomogeneity of the Fe/AC adsorbent sample is observed. When you enlarge the image, you can see conglomerates of different sizes and shapes but also metallic oxide clusters, as well as coal pores of different shapes and sizes: elongated, cylindrical, or pyramidal.

An obvious inhomogeneity of the structures, as well as different shapes of the particles and the presence of metal oxide clusters of different sizes, can be observed at the Zn/AC adsorbent.

### 2.2. Textural Characteristics

**Figure 2:**
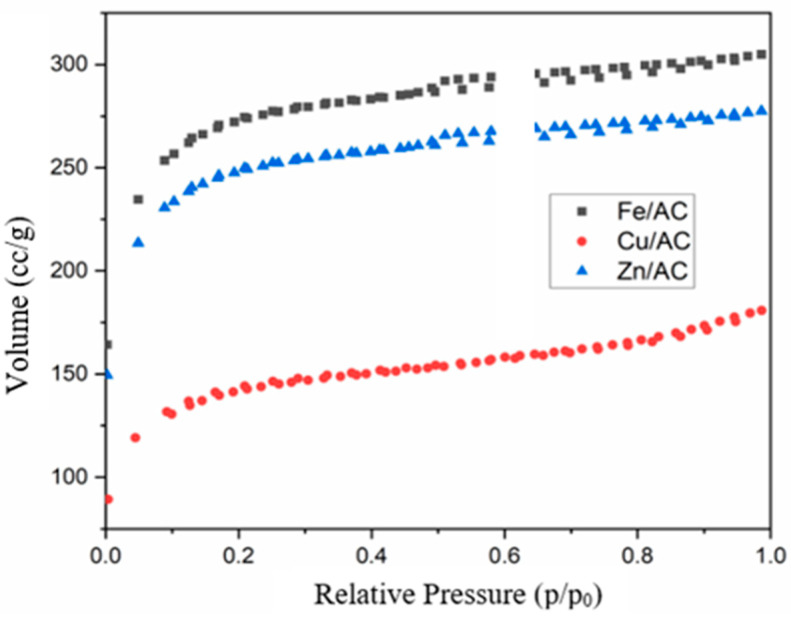
N_2_ adsorption-desorption isotherms of adsorbents.

Textural parameters of the prepared adsorbents were evaluated using the adsorption/desorption isotherms recorded at relative pressures p/p_0_ ranging from 0.005 to 1.0 (Figure 2).

The Cu/AC adsorbent shows a type I adsorption-desorption isotherm with an H IV hysteresis loop specific to materials with cylindrical pores, the hysteresis loop being very narrow as can be seen in Figure 2. Thus, the adsorption and desorption curves practically overlap over almost the entire range of vapor pressure variation studied.

**Figure 3:**
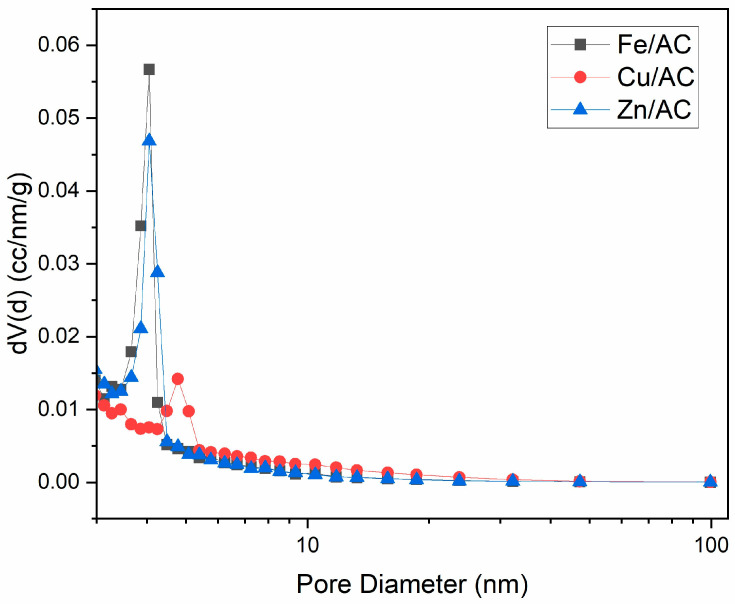
Derivative of the pore size distribution for Fe/AC, Cu/AC and Zn/AC adsorbents.

The pore size distribution was highlighted by graphically representing the derivative of the curve expressing the pore volume of the adsorbents as a function of the pore diameter (Figure 3). The pore size distribution curve of the Cu/AC adsorbent (Figure 3) shows a high concentration of mesopores and a low concentration of macropores. The structure is predominantly mesoporous, with a peak occurring at pore diameters greater than 4 nm.

The Fe/AC adsorbent exhibits a type I adsorption-desorption isotherm featuring a narrow hysteresis loop of type H IV, which is typical for materials with cylindrical pores (Figure 2). In this instance, the adsorption and desorption curves closely overlap across nearly the entire range of vapor pressure variations studied.

The pore size distribution curve for the Fe/AC adsorbent exhibits a narrow distribution with a high content of mesopores (Figure 3). The structure is primarily mesoporous, with the peak located at pore diameters greater than 2 nm.

The adsorption and desorption isotherm for the Zn/AC adsorbent shows that the adsorption and desorption curves closely overlap across nearly the entire range of vapor pressure variations studied, resulting in a very narrow hysteresis loop (Figure 2). Additionally, the isotherm exhibits linearity for relative pressure values greater than 0.2. The adsorption-desorption isotherm is classified as type I, featuring an H4 hysteresis loop, which is characteristic of materials with cylindrical pores and a narrow pore size distribution. The pore size distribution curve for the Zn/AC adsorbent reveals a narrow range of pore sizes, predominantly consisting of mesopores, with a peak in pore diameter values greater than 2 nm (Figure 3).

Both Fe/AC and Zn/AC adsorbents exhibit specific surface areas exceeding 800 m²/g, demonstrating quite similar values. In contrast, the specific surface area of the Cu/AC adsorbent is nearly half that of the other two. Additionally, the total pore volumes for both the Fe/AC and Zn/AC adsorbents align closely and are significantly larger than that of the 20% Cu/AC adsorbent. Furthermore, the average pore diameter for the Fe/AC and Zn/AC adsorbents is smaller than that of the Cu/AC adsorbent (see Table 1). The specific surface area values and average pore diameter of the prepared metal adsorbents indicate that diffusional barriers to the adsorption processes of lower mercaptans are excluded. This suggests that the size of the metal clusters deposited on the granular activated carbon support does not pose any risk of diminishing butanethiol adsorption capacity, nor will it adversely affect the adsorption performance of the prepared adsorbents.

### 2.3. Thermogravimetric Analysis

**Figure 4:**
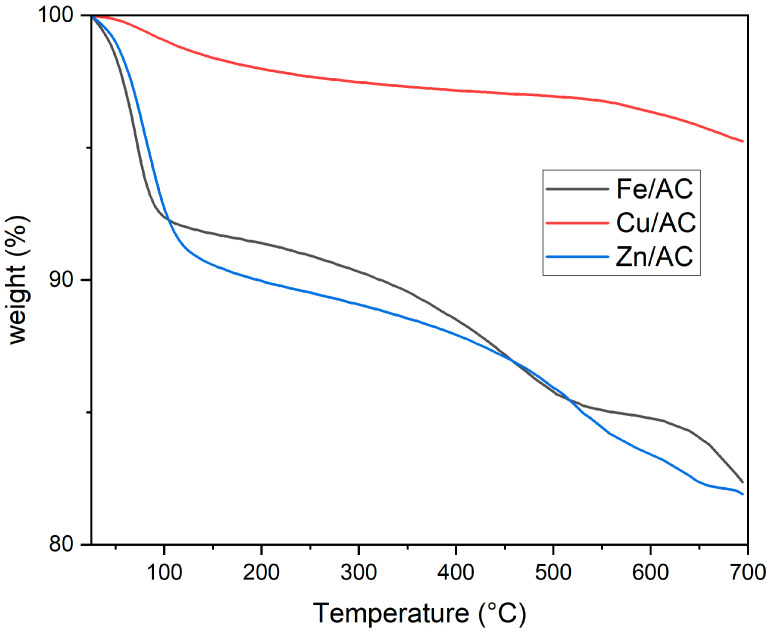
Thermogravimetric analysis of adsorbents.

TG analysis of adsorbents applied to granulated carbon indicated that Fe and Zn-based adsorbents exhibited higher mass losses compared with the copper-based adsorbent (Figure 4). For the copper-based adsorbent, the initial segment of the TGA curve (between 40 °C and 200 °C) showed greater mass losses attributed to the evaporation of volatile components, such as water and potential traces of organic compounds from the activated carbon. During the subsequent heating phase, from 200 °C to 500 °C, mass losses decreased, likely due to the ongoing thermal decomposition process of Cu hydroxide, which apparently was not fully completed during the adsorbent’s calcination. In the third stage (500 °C to 700 °C), the mass loss curve exhibited a steeper slope compared with the previous phase. This mass loss may be linked to the decomposition of certain classes of organic compounds within the activated carbon support that demonstrate greater thermal stability, such as carbenes or graphite precursors. Overall, these findings highlight the significant thermal stability of the copper-based adsorbent.

In the case of Fe and Zn-based adsorbents, two distinct zones of mass loss variation with increasing temperature can be identified. In the first zone, which extends up to approximately 140 °C, the mass losses are greater compared with the Cu-based adsorbent, indicating a higher content of volatile components, most likely water. The second heating phase, ranging from around 140 °C to 700 °C, exhibits a relatively constant slope in the mass variation curve for both types of adsorbents. Consequently, the mass losses in this region are lower than those observed in the first zone, likely resulting from the ongoing thermal decomposition of the hydroxides of the two metals, suggesting incomplete calcination of the adsorbents. It is evident that, for these adsorbents, the elimination of water generated during calcination is more challenging than with the Cu-based adsorbent. This behavior can be attributed to the slower diffusion of water molecules within the pores of the Fe and Zn adsorbents, likely due to stronger interactions between the water molecules and the respective metal oxides. At temperatures exceeding 450 °C, mass losses may also arise from the cracking reactions of certain organic compounds that are typically present in the pores of activated carbons (commonly referred to as tars). This cracking process is catalyzed by superacids. Thus, the superacid structures formed by the reactions of Zn and Fe oxides (acting as Lewis acids) in the presence of trace amounts of water (acting as a Bronsted acid) facilitate the cracking of the organic compounds within the activated carbon’s pores, leading to the generation of volatile organic compounds that contribute to mass losses.

### 2.4. FTIR Analysis

**Figure 5:**
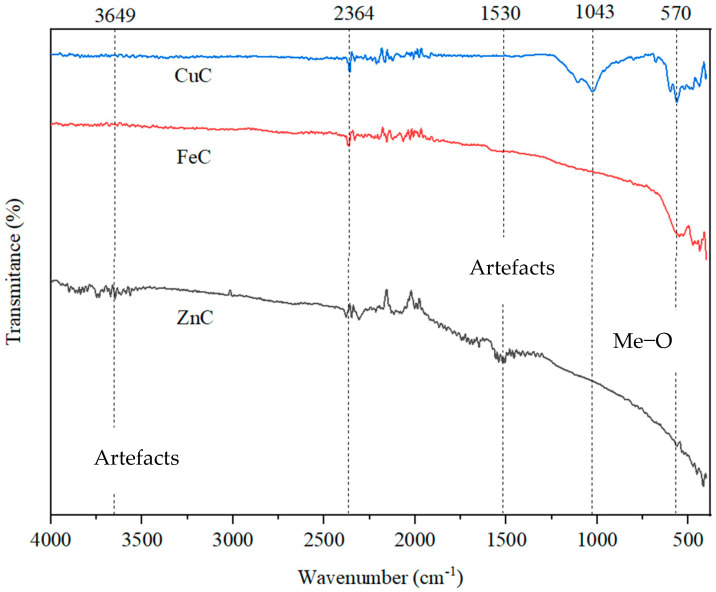
FTIR spectra of Cu/AC, Fe/AC, and Zn/AC adsorbents.

The three samples of biochar doped with metal oxides were analyzed by FTIR, in order to highlight the presence of functional groups and chemical bonds (Figure 5). The vibrations in the region of 570 cm⁻¹ are attributed to metal-oxygen bonds. 

The spectrum of the sample treated with Cu shows a distinct band at the wave number 1043 cm⁻¹, which is attributed to C-O bonds specific to phenols and alcohols. This observation indicates the preservation of the oxygenated functional groups of carbon. The presence of these groups enhances the surface activity in catalytic applications [19]. The peaks observed between 400–700 cm^−1^ correlate to metal oxide bonds [20,21]. In our study, the characteristic peak at 570 cm^−1^ with Cu-O stretching vibrations confirmed the formation of CuO [22]. The peak at 1100 cm^−1^ corresponds to Cu-OH stretching vibrations, indicating the presence of copper (II) oxide [21].

Bands below 570 cm⁻¹ are more intense for the ZnC material, suggesting better coverage of carbon due to zinc’s higher affinity for the oxygen atoms. Conversely, the CuC assembly displays weaker interactions with the support material, resulting from a lower number of Cu-O associations. This behavior correlates with the pronounced band at 1043 cm⁻¹, which indicates the oxygen atoms remaining in the structure of the functional groups. The moderate interactions of copper with oxygen are attributed to a finer distribution of the metal on the support, a conclusion supported by the smaller pore volume highlighted in the textural analysis [23,24].

The spectrum of the sample treated with zinc chloride shows a wide band but of weak intensity in the area 3500–3650 cm^−1^, representing stretching vibration of O-H and can be assigned to water vapor absorption, but also to water in the condensed state. In a similar situation, the band in the area 1500–1650 cm^−1^ can be associated with C=C stretching vibration in alkene or aromatics, but there is possibly overlapping with the rotational and bending vibrational bands of the water vapor (the so-called vibration-rotation bands of H_2_O vapor) [25,26,27,28]. Moreover, it must be noted that the C=C stretching vibrations are low polar and low active in IR absorbance. For this sample, only characteristic bands between 450 and 650 cm^−1^ related to Zn–O, were highlighted, confirming the presence of a metal oxide group on the surface of the analyzed material [29]. For the 1546 cm^−1^ band, as Saussey et al. mentioned [30], it is difficult to assess the origin of this signal, characteristic for the species reversibly adsorbed at room temperature and that tends to disappear with time.

In the case of FeC sample can be seen some broad peaks with maximum at 570 and about 480 cm^−1^, indicating the presence of the metal oxides with different oxidation number, FeO or a-Fe_2_O_3_ [31,32]. These findings are in alignment with the results of the XRD analysis, which enabled the identification of the two distinct species of iron oxides.

### 2.5. XRD Analysis

**Figure 6:**
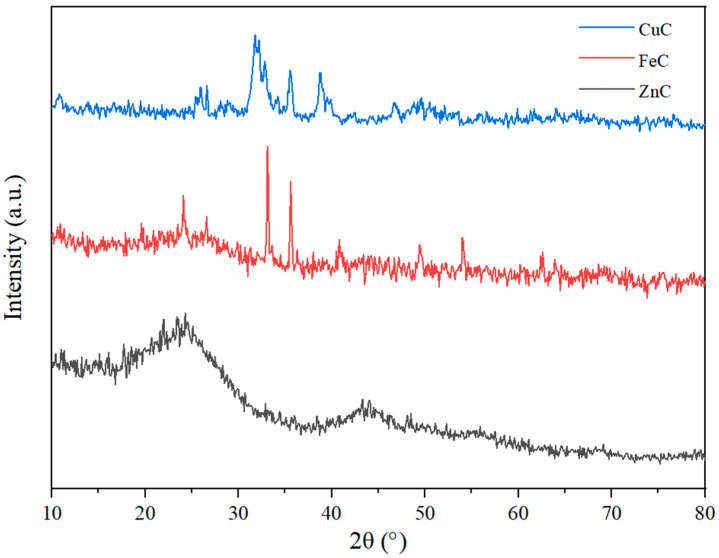
X-ray diffraction spectrum of Cu/AC, Fe/AC, and Zn/AC adsorbents.

Specific peaks for compounds based on copper, lignin, and graphite were identified on the diffraction spectrum of the Cu/AC sample (Figure 6). Thus, copper oxide (CuO) (ICDD file 073-6023) shows specific peaks at 35.58 (11), 38.74 (111), 49.25° (02), 61.50° (13), 65.86 (022), and 72.30° (311). Moreover, this result agrees with the FTIR data showing the presence of this species. Copper sulfide (ICDD file 065-3561) shows a high-intensity peak at 32° (103), accompanied by other less intense peaks at 29.48 (102), 48.30 (110), and 59.80 (116). The lignin identified in the sample shows small but well-individualized peaks specific to planes (101) at 16.52°, (203) at 18.30°, and (200) at 22.80° [33]. The sample also shows an appreciable content of graphite (ICDD file 056-0159) reflected by the peaks at 26.54° (plane 002) and at 42.34° (plane 100), corresponding to a higher degree of lignin crystallinity (15.60%).

The diffraction spectrum of the Fe/AC sample (Figure 6) shows well-individualized peaks (2θ) of iron compounds represented by iron oxide (Fe_2_O_3_) (ICDD file 073-8433) at 24.10º (012), 33.12º (104), 35.66º (110), 40.80º (113), 49.50 (024), 54º (116), 62.40º (214), and 64º (300), [34] and, respectively, by atomic Fe (ICDD file 006-0696) at 44.68º (plane 011) and 65º (220) corresponding to a high iron content in the sample. These results are in accord with FTIR findings. The amorphous character of lignin is highlighted by the broad peak in the interval 14–30º 2θ, within which the peak of the (200) plane of lignin/biomass is noticeable. The partial graphitization of lignin is also demonstrated by the specific graphite peaks at 26.54º (plane 002) and 54.66° (plane 004), a reduced proportion of lignin passing into graphite (5.91%) by applying the thermal treatment. The increase in the degree of crystallinity of the lignin in the sample (16.24%) is noticeable compared with the Cu/AC sample.

The diffraction spectrum of the Zn/AC sample (Figure 6) is dominated by the broad peak in the range 14–32º 2θ, within which the peak of the (200) plane of lignin/biomass at 24º 2θ, corresponding to an amorphous behavior of the biomass, is also noticeable, accompanied by other smaller peaks at 18º and 22º 2θ [33]. A thin peak at 26.54º 2θ of the (002) plane indicates a weak graphitization of the biomass. The other peaks identified on the spectrum of the Zn/AC sample belong to zinc compounds, both atomic zinc (ICDD file 004-0831) through peaks 36.24º (002), 39º (100) and 43.24º (101), and zinc oxide (ICDD file 075-1533) through peaks 30.72º (100), 34.32º (002), 35.60º (101), 46.74º (102) and 48.50 (110). Moreover, the presence of zinc oxide is also supported by the results of FTIR analyses. The increase in the proportion of lignin in the sample (59.16%) and the degree of crystallinity of the lignin (18.68%) proportional to the peak area of the plane (200) of the lignin/biochar can be noted. The Zn/AC sample shows the lowest degree of graphitization of lignin among all the samples analyzed, a phenomenon probably explained by the high degree of adsorption of Zn atoms on the surface of the planes of carbon atoms that led to the increase in the degree of thermal stability of biomass/lignin [35].

Based on the experimental data and the chosen structural model, the average size of the crystallites was estimated, and the degree of crystallinity of the metal phase *X_c_* was calculated (Table 2) using the following equation:

$$X_{c}=\frac{\sum A_{crys}}{\sum A_{crys}+A_{amorph}}$$

*A_crys_* is the adjusted area of the crystalline phase, and *A_amorph_* is the adjusted area of the amorphous phase.

It can be seen from Table 2 that the metals present in the three adsorbents are found both in the oxide form and in the metallic form. The formation of the metallic phase is due to the reduced activity of the carbohydrates present in the grape seeds. The average size of the crystallites is larger in the case of the Fe/AC adsorbent compared with the other two and smaller in the case of the Zn/AC adsorbent. The degree of crystallinity has values over 96% for both the metal phase and the oxide phase in the case of the Cu/AC adsorbent. In the other two adsorbents, the degree of crystallinity varies over a wider range in the metallic phase compared with the oxide phase. Thus, in the Fe/AC adsorbent, the oxide phase presents a degree of crystallinity of approx. 80%, and in the Zn/AC adsorbent, the metallic phase shows a degree of crystallinity of approx. 90%.

### 2.6. Butanethiol Adsorption Tests on Prepared Adsorbents

The adsorbents were kept in the oven at 130 °C for 24 h and then kept in the desiccator until they were loaded into the absorption reactor. The operating parameters of the reactive adsorption plant were the following:

(a) Adsorbent mass: 40 g;
(b) Gas flow (nitrogen): 300 cm^3^/min;
(c) Butanethiol concentration in the carrier gas: 121.11 g/m^3^;
(d) Adsorber temperature: 150 °C.

**Figure 7:**
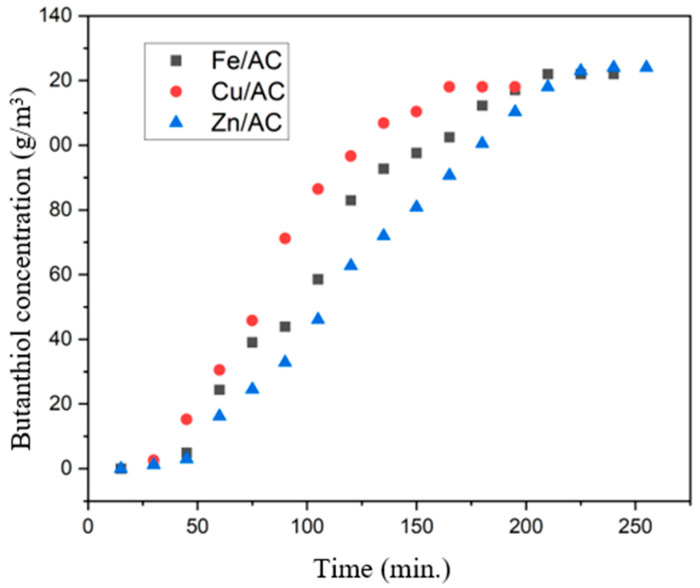
Variation of butanethiol concentration in nitrogen with contact time during adsorption on adsorbents at atmospheric pressure and 150 °C.

The variation of the 1-butanethiol concentration in the nitrogen flow discharged from the bubbler, with the contact time in the case of the adsorption experiment on the Cu/AC adsorbent, is presented in Figure 7.

The adsorption capacity of butanethiol on the tested adsorbents was calculated using the method of integrating the area up to the moment of penetration of the adsorbent layer, applying the method presented by Deng et al. [36].

From the analysis of the reactive adsorption curve of butanethiol on 20% Cu/AC, it follows that after 80 min of experimentation the adsorbent is saturated in a proportion of approx. 50%. It is observed that the total saturation of the adsorbent occurs after approximately 165 min from the start of the experiment.

By integrating the adsorption curve up to the moment of saturation of the absorbent (min. 165), the amount of 9368.79 g/m^3^ butanethiol adsorbed at a contact time of 165 min results. By reference to the amount of absorbent (40 g), the maximum adsorption capacity is obtained at 1.696 cm^3^ butanethiol/g adsorbent Cu/AC.

From the analysis of the reactive adsorption curve of butanethiol on 20% Fe/AC, it follows that after 105 min of exposure the adsorbent is saturated in a proportion of approx. 50%. It was observed that the total saturation of the adsorbent occurs approximately 210 min after the exposure to the pollutant (Figure 7).

By integrating the adsorption curve up to the moment of saturation of the absorbent (min. 210), the value 12,579.42 g/m^3^ butanethiol adsorbed during 210 min results. By reference to the amount of absorbent (40 g), the maximum adsorption capacity is obtained at 1790 cm^3^ butanethiol/g adsorbent 20%Fe/AC.

From the analysis of the reactive adsorption curve of butanethiol on Zn/AC, it follows that after 150 min of exposure, the adsorbent is saturated in a proportion of approx. 50%. It was observed that the total saturation of the adsorbent occurs after approximately 255 min of exposure to the pollutant (Figure 7).

By integrating the adsorption curve up to the moment of saturation of the absorbent (min. 255), the value 13,662.28 g/m^3^ butanethiol adsorbed for 255 min results. By reference to the amount of absorbent (40 g), the maximum adsorption capacity is obtained 1.601 at cm^3^ butanethiol/g adsorbent Zn/AC.

Feedback: The adsorption capacity for the studied adsorbents is presented in Table 3. It is observed that the adsorption capacity is not proportional to the specific surface of the adsorbents. Thus, the Cu/AC adsorbent has a 1-butanethiol adsorption capacity close to that of the Fe/AC adsorbent (approx. 5% lower than it), although the specific surface area is almost half that of the Fe/AC adsorbent. The Zn/AC adsorbent has a lower 1-butanethiol adsorption capacity by approx. 10% compared with that of the Fe/AC adsorbent, although both adsorbents have a similar specific surface (the Fe/AC adsorbent has a specific surface approx. 2% higher than that of the Zn/AC adsorbent). It can be considered that the adsorption capacity of adsorbents with a large specific surface area is mainly influenced by the nature and reactivity of the metal oxide clusters deposited in the pores of the adsorbent.

Recent studies [12] have investigated the chemical interactions between 1-butanethiol and metal clusters of Fe and Cu deposited on various supports, revealing similar activation energy values specific to chemisorption reactions. Given that Zn exhibits a sulfur affinity comparable to that of Fe and Cu, it is reasonable to expect that Zn-based clusters will also demonstrate similar activation energy values. This suggests that the adsorption of 1-butanethiol on these clusters is irreversible. The absence of butenes in the effluent from the adsorber indicates that chemisorption occurs at the operational temperatures, highlighted by the interaction between the Bronsted acid of the thiol and the Lewis acids present in the metal oxides (Zn, Fe, and Cu). This interaction results in the formation of a superacid structure that remains stable under these conditions. Consequently, regenerating these saturated adsorbents requires thermal treatment to desorb butenes produced during desulfurization, followed by regeneration of the adsorbent via oxidation with atmospheric oxygen or reduction in a hydrogen stream. Both of these processes are highly exothermic and create a significant local thermal effect due to the low thermal conductivity of the activated carbon support, which can promote the sintering of metal clusters. As a result, the regeneration of these adsorbents and their long-term stability are constrained. Increasing the number of operational cycles necessitates enhancements to the conductivity of the activated carbon-based adsorbent, potentially achieved by introducing a second metal with high thermal conductivity, such as metallic copper.

## 3. Materials and Methods

### 3.1. Materials

The reagents used in this study included iron(II) chloride tetrahydrate (ReagentPlus^®^, 98%), copper(II) sulfate pentahydrate (ACS reagent, ≥98.0%), zinc chloride (reagent grade, ≥98%), Pluronic P-123, and ammonia solution (25% for analysis), all of which were sourced from Aldrich-Sigma (Schnelldorf, Germany). The activated carbon utilized was of the granular cylindrical type (code CA0346, Scharlau, Barcelona, Spain).

### 3.2. Synthesis of Adsorbent

The adsorbents were synthesized using the conventional precipitation method, which involved one or more steps and the addition of an anti-clumping additive. The anti-clumping additive Pluronic P123 is added to improve the texture, morphology, and pore size of the metal oxide clusters that form during the precipitation step. Also, due to its hydrophobic characteristics, it reduces the tendency of the clusters to agglomerate during the precipitation process, reducing the risk of clogging the pores of the activated carbon support [19]. The amounts of precursors and the number of impregnation stages were calculated to ensure that the concentration of the three metals deposited on the adsorbents was 20% by weight. The adsorbent precursors were dissolved in 100 mL of distilled water at a temperature of 60 °C, with magnetic stirring at a speed of 300 rpm. To this mixture, 0.5 g of Pluronic P-123 and 50 g of granulated coal were added. A 25% aqueous ammonia solution was then introduced while stirring at 600 rpm until the pH reached 9. Subsequently, the mixture was placed in a ventilated oven and heated in two stages: the first stage at 60 °C for 72 h, followed by a second stage at 160 °C for 3 h. After heating, the adsorbent was washed three times with distilled water. The activation of the three adsorbents was completed by calcining them in an oven at 450 °C for 4 h under a protective layer of inorganic powders.

### 3.3. Characterization Methods

The three prepared adsorbents were characterized using various techniques, including electron microscopy (SEM, Scios 2 HIVAC Dual-Beam FIB-SEM with ultra-high resolution; Thermo Fisher, Brno, Czech Republic), textural analysis, thermogravimetric analysis (TGA), FTIR, and XRD analyses. The microstructural morphologies of the adsorbents were examined with a FEI Inspect Scanning Electron Microscope (SEM) operating under vacuum conditions, with an energy range of 200 V to 30 kV.

Textural characteristics were determined using a NOVA 2200e gas sorption analyzer (Quantachrome Tools, Boynton Beach, FL, USA). Nitrogen adsorption/desorption isotherms were recorded at 77.35 K, with a relative pressure range (p/p_0_) between 0.005 and 1.0. The obtained data were processed using NovaWin software (version 11.03). The specific surface area was calculated using the standard BET equation (Brunauer-Emmett-Teller), while the total pore volume was estimated based on the desorbed volume at a relative pressure close to unity, applying the Barrett-Joyner-Halenda (BJH) method. The pore size distribution and mesopore volume were derived from the desorption branch of the isotherm using the BJH model. Prior to the adsorption measurements, the samples were degassed at 160 °C under vacuum for four hours. Thermogravimetric analyses of the three adsorbents were conducted using a TGA/IST (Setaram Labsys Evo S60/58986 TG analyzer (Cranbury, NJ, USA)) in a nitrogen atmosphere, over a temperature range of 25 to 700 °C. For the qualitative analysis of the materials, the Fourier Transform Infrared (FTIR) spectroscopy with Attenuated Total Reflection (ATR) technique was performed. This analysis was performed in order to analyze the structure, including functional groups. It used the FTIR spectrometer Shimadzu IRAffinity-1S (Shimadzu, Kyoto, Japan), equipped with the GladiATR-10 accessory. Measurements were performed in a wavenumber region of 380 to 4000 cm⁻¹, with a spectral resolution of 4 cm⁻¹.

X-ray diffraction (XRD) analyses were carried out using a D8 Advance diffractometer (Bruker-AXS, Karlsruhe, Germany) with a copper anode X-ray tube (Cu-Kα radiation, λ = 1.54 Å), θ-θ configuration, and Bragg–Brentano geometry. The measurements were taken within a range of 10° to 80° (2θ), with the following parameters: voltage of 40 kV, anode current of 40 mA, scan step of 0.1°, and scan speed of 0.1°/5 s. The raw data from the measurements were obtained using the XRD Commander software v3.1. Qualitative interpretations were made using Diffracplus EVA v.14 software alongside the PDF-ICDD database. For quantitative analysis, the Rietveld technique was employed using TOPAS 4.1 software, and peak matching was performed utilizing the pseudo-Voigt profile function.

**Figure 8:**
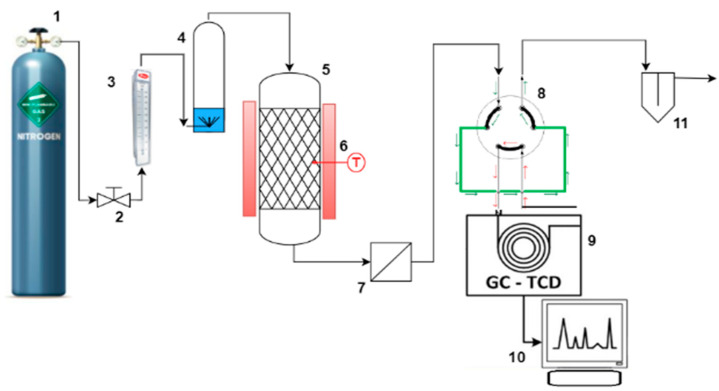
Scheme of the experimental adsorption unit (1. gas cylinder with pressure regulator; 2. flow control valve; 3. rotameter-flow indicator; 4. bubbler; 5. adsorption reactor; 6. thermostatic heating mantle with thermoregulator and thermocouple; 7. filter; 8. gas sampling valve with 2 cm³ loop; 9. gas chromatograph with TCD thermal conductivity detector; 10. recorder; 11. hydraulic closing).

The 1-butanethiol adsorption experiments were conducted in a continuous system with a fixed adsorbent layer at atmospheric pressure and a temperature of 150 °C. The experimental setup included a pressurized gas cylinder (nitrogen), a pressure reducer, a flow regulator, a rotameter for measuring gas flow, a bubbler, a thermostated adsorption reactor with an electric heating jacket, and a GCHF 18.3 gas chromatograph (GC/MS Agilent: GC 7890A with 5975 MSD Triple Quad—Agilent Technologies, Inc., Santa Clara, CA, USA), acting as a process analyzer with a thermal conductivity detector (TCD) (Figure 8).

The carrier gas used to transport 1-butanethiol in the gas phase was nitrogen, with a flow rate adjusted to 300 mL/min. To determine the concentration of 1-butanethiol at the entrance of the reactor, we monitored the weight changes of a bubbling vessel filled with 1-butanethiol during the nitrogen bubbling process. Periodic weight measurements of the bubbling vessel were conducted using a Kern KB2500-2N, (Kern, Baden-Württemberg, Germany) analytical balance. The time-based variation of butanethiol concentration at the exit of the adsorption reactor was analyzed using gas chromatography on a GCHF 18.3 process gas chromatograph.

## 4. Conclusions

New adsorbent materials for removing 1-butanethiol from a gaseous stream were prepared, using active carbon doped with Cu, Fe, and Zn oxides.

SEM analysis of the synthesized adsorbents revealed a uniform distribution of copper oxide clusters. In contrast, the zinc oxide clusters exhibited a less uniform size and distribution compared with the other two adsorbents. These variations primarily result from the influence of the anti-clumping additive Pluronic P-123 on the precipitation process of copper, iron, and zinc salts utilized in this study. The liquid nitrogen adsorption-desorption isotherm, obtained following textural analysis, demonstrated analogous behavior among the three adsorbents during the liquid nitrogen adsorption-desorption process, characterized by very narrow hysteresis loops. The pore size distribution curves indicated a significant presence of mesopores in all three prepared adsorbents. The specific surface areas of the zinc/activated carbon (Zn/AC) and iron/activated carbon (Fe/AC) adsorbents were considerably greater than that of the copper/activated carbon (Cu/AC) adsorbent. This phenomenon correlates with the markedly higher mass loss observed in the latter two adsorbents at temperatures exceeding 450 °C, a loss facilitated by the cracking reactions of tar within the pores of these adsorbents in the presence of the metal oxide Lewis acids. 

TGA analysis of the three adsorbents indicated differing volatile content and varying mass loss values. The observed mass losses are attributed to the thermal decomposition of specific compounds present in the compositions of the three adsorbents. The elevated values of volatile content and mass losses in the cases of Fe/AC and Zn/AC adsorbents are likely due to the enhanced thermal stability of intermediates formed during the decomposition of oxy-hydroxides obtained from the precipitation of chloride-type salts, as well as the cracking activity of the metal oxide-type Lewis acids. Furthermore, the results of FTIR and XRD analyses are congruent, confirming the presence of metal oxide groups on the surfaces of the analyzed materials. 

The adsorption capacity of 1-butanethiol on the prepared adsorbents is influenced by their structural characteristics and specific surface areas but is particularly affected by the size of the metal oxide clusters deposited within the pores of each adsorbent. Consequently, the adsorbent consisting of Fe/AC, which possesses larger crystallite sizes, demonstrates a higher adsorption capacity compared with the other two adsorbents. In contrast, the adsorbent based on Zn/AC, which features smaller crystallite sizes, exhibits a lower adsorption capacity than its counterparts. The regeneration of adsorbents such as activated carbon necessitates the incorporation of dual components via doping. It is advisable for one of these components to be a metal with high thermal conductivity, such as metallic copper (Cu), in order to mitigate the sintering of these clusters that may occur due to localized temperature increases.

## Figures and Tables

**Table 1:** Textural characteristics for adsorbents Cu/AC, Fe/AC, and Zn/AC.

No.	Adsorbent	Specific Surface Area (m^2^/g)	Total Pore Volume (cm^3^/g)	Average Pore Diameter (nm)
1	Cu/AC	443.7	0.2797	2.521
2	Fe/AC	836.1	0.4717	2.256
3	Zn/AC	816.2	0.4627	2.268

**Table 2:** The average size of the crystallites and the degree of crystallinity of the metallic phases.

Sample	Compound	Crystallite Size (nm)	Crystallinity Degree, *X_c_* %
Cu/AC	copper oxide	124.7	96.37
	copper sulphide	82.4	96.35
	copper	54.7	99.09
Fe/AC	iron oxide	35.01	80.72
	iron	85.41	99.21
Zn/AC	zinc	56.6	90.05
	zinc oxide	97.4	98.94

**Table 3:** Adsorption capacity for the studied adsorbents.

No.	Adsorbent	Amount Adsorbed (g Butanethiol/m^3^)	Maximum Adsorption Capacity (cm^3^ Butanethiol/g Adsorbent)
1	Cu/AC	9368.79	1.696
2	Fe/AC	12,579.42	1.790
3	Zn/AC	13,662.28	1.601

## Data Availability

The original contributions presented in this study are included in the article. Further inquiries can be directed to the corresponding author.
